# IL-2Rα up-regulation is mediated by latent membrane protein 1 and promotes lymphomagenesis and chemotherapy resistance in natural killer/T-cell lymphoma

**DOI:** 10.1186/s40880-018-0334-8

**Published:** 2018-10-19

**Authors:** Liang Wang, Xi-wen Bi, Yu-jia Zhu, Ying-zhi He, Qiu-yu Lai, Zhong-jun Xia, Qing-qing Cai

**Affiliations:** 1 Department of Hematology, ZhuJiang Hospital, Southern Medical University, Guangzhou, 510280 Guangdong P. R. China; 2 State Key Laboratory of Oncology in South China, Collaborative Innovation Center for Cancer Medicine, Guangzhou, 510060 Guangdong P. R. China; 3 Department of Medical Oncology, Sun Yat-sen University Cancer Center, Guangzhou, 510060 Guangdong P. R. China; 4 Department of Radiation Oncology, Sun Yat-sen University Cancer Center, Guangzhou, 510060 Guangdong P. R. China; 5 Department of Hematologic Oncology, Sun Yat-sen University Cancer Center, Guangzhou, 510060 Guangdong P. R. China

**Keywords:** Natural killer/T-cell lymphoma, Latent membrane protein 1, Epstein–Barr virus, Interleukin-2 receptor alpha

## Abstract

**Background:**

Natural killer/T-cell lymphoma (NKTCL) is a highly aggressive non-Hodgkin lymphoma often resistant to chemotherapy. Serum level of soluble IL-2 receptor α (IL-2Rα) is elevated in NKTCL patients and correlates significantly with treatment response and survival. In the current study we examined the potential role of IL-2Rα by over-expressing IL-2Rα in representative cell lines.

**Methods:**

Levels of IL-2Rα were evaluated in the human natural killer cell line NK-92 and the NKTCL cell line SNK-6. Lentiviral vectors were used to express latent membrane protein 1 (LMP1) in NK-92 cells, and IL-2Rα in both NK-92 and SNK-6 cells. The biological effects of these genes on proliferation, apoptosis, cell cycle distribution, and chemosensitivity were analyzed.

**Results:**

Expression of IL-2Rα was significantly higher in SNK-6 cells than in NK-92 cells. Expressing LMP1 in NK-92 cells remarkably up-regulated IL-2Rα levels, whereas selective inhibitorss of the proteins in the MAPK/NF-κB pathway significantly down-regulated IL-2Rα. IL-2Rα overexpression in SNK-6 cells promoted cell proliferation by altering cell cycle distribution, and induced resistance to gemcitabine, doxorubicin, and asparaginase. These effects were reversed by an anti-IL-2Rα antibody.

**Conclusions:**

Our results suggest that LMP1 activates the MAPK/NF-κB pathway in NKTCL cells, up-regulating IL-2Rα expression. IL-2Rα overexpression promotes growth and chemoresistance in NKTCL, making this interleukin receptor a potential therapeutic target.

**Electronic supplementary material:**

The online version of this article (10.1186/s40880-018-0334-8) contains supplementary material, which is available to authorized users.

## Background

Natural killer/T-cell lymphoma (NKTCL) is an aggressive non-Hodgkin lymphoma, with no standard treatment [1, 2]. Due to the over-expression of multidrug resistance genes, NKTCL is typically resistant to anthracycline-based chemotherapy (e.g., CHOP regimen) [3–5]. l-Asparaginase or pegaspargase have improved patient prognosis and thus increasingly used as first-line treatments in recent years [6–9]. Asparaginase-based regimens still fail in 20%–40% of the patients [6, 7, 9, 10], and few second-line treatments have been described for asparaginase-refractory cases [11].

Interleukin-2 (IL-2) is a pro-inflammatory cytokine that activates and maintains immune responses [12]. IL-2 exerts its biological effects by binding to its receptor IL-2R on the cell surface. IL-2R consists of three subunits. The α subunit, also known as CD25 or Tac antigen, does not participate on its own in IL-2-triggered signal transduction due to its very short intracellular domain. When the α subunit combines with IL-2Rβ and IL-2Rγ, the affinity of IL-2R to IL-2 increases. Proteolytic cleavage of membrane-bound IL-2Rα generates a soluble form of IL-2Rα (sIL-2Rα) [13]. In a variety of solid malignant tumors as well as hematologic malignancies, serum sIL-2Rα is elevated and correlates with poor patient prognosis [14–18]. In a previous retrospective analysis from this research group, we reported elevated serum sIL-2Rα in NKTCL patients than in healthy individuals [19]. We also found significant correlation between higher sIL-2Rα with poor chemotherapy response and patient prognosis [19].

In the present study, we examined the potential effects of IL-2Rα over-expression on cell proliferation, cell cycle distribution and sensitivity to chemotherapeutic drugs in representative NKTCL cell lines. Mechanistic investigation identified latent membrane protein 1 as a down-stream effector.

## Methods

### Cell lines and culture

The human NKTCL cell line SNK-6 and human natural killer cell line NK-92 (both were generously donated by professor Zhang Ming-zhi from the First Affiliated Hospital of Zhengzhou University) were incubated at 37 °C in an atmosphere of 5% CO_2_. SNK-6 cells were cultured in RPMI 1640 (Hyclone, USA) supplemented with 10% fetal bovine serum (FBS; Gibco, USA) and recombinant human IL-2 (1000 U/ml; Sigma, USA). NK-92 cells were maintained in RPMI 1640 containing 10% FBS.

### Construction of cell lines overexpressing LMP1 and IL-2Rα

Lentivirus vectors expressing latent membrane protein-1 (LMP1) were constructed as described previously [20]. Briefly, full-length IL-2Rα cDNA was inserted into the *Not*I/*Bam*HI sites of the LV5 vector (GenePharma, China). Vector encoding IL-2Rα and the packaging vectors pGag/Pol, pRev, and pVSV-G (GenePharma) were co-transfected into HEK293T cells using Lipofectamine 2000 (Beyotime, Shanghai, China). Supernatant containing recombinant lentivirus was collected 72 h after transfection. NK-92 and SNK-6 cells (1 × 10^4^ cells/well) were separately infected for 24 h with the viruses in the presence of polybrene (5 μg/ml). The medium was replaced with fresh medium, and cells were cultured another 48 h. Transfected cells were selected using puromycin (1.0 μg/ml) for 7–10 days prior to expansion.

### Western blot analysis

Western blot analysis was performed as described previously [20]. Briefly, cells were washed with ice-cold PBS and suspended in radioimmunoprecipitation assay (RIPA) lysis buffer (Biyuntian Biotech, Shanghai, China) containing 1% phenylmethylsulfonylfluoride. After centrifugation at 18,735×*g* for 10 min at 4 °C, the supernatant (10 μg protein per lane) was fractionated using 12% SDS-PAGE and transferred to polyvinylidene fluoride (PVDF) membranes (Millipore, Billerica, MA, USA). The membranes were incubated at room temperature for 1 h with one of the following primary antibodies: IL-2Rα, B-Raf, p-B-Raf, p38, p-p38, ERK, p-ERK (Abcam, Shanghai, China); LMP1, JNK, p-JNK (Santa Cruz, Shanghai, China); p65 and cyclins A1, A2, B1, and D (Boster); cyclin E (Proteintech, Wuhan, China); cyclin-dependent protein kinase (CDK) 1 (Abcam); and CDK2 and 4 (Boster). After extensive washing, the membranes were incubated with a horseradish peroxidase-conjugated goat anti-rabbit IgG (1: 20,000; Boster, Wuhan, China) at room temperature for 40 min. Signals were detected with an enhanced chemiluminescence kit (Amersham Pharmacia, Piscataway, NJ, USA). Results were normalized against glyceraldehyde-3-phosphate dehydrogenase (GAPDH). Sample protein concentration was determined using a BCA method.

### Quantitative real-time PCR analysis

Quantitative RT-PCR was performed as described previously [20]. Briefly, total RNA was isolated using Trizol (Invitrogen, USA). Total RNA (1 μg) was reverse-transcribed into cDNA using the Bestar™ qPCR RT Kit (DBI Bioscience, China). The qRT-PCR reaction was conducted in a total volume of 20 μl containing 10 μl DBI Bestar^®^ SybrGreen qPCR Master Mix (DBI Bioscience), cDNA derived from 0.2 μg of input RNA, 5 pM of each primer, and 7 μl double-distilled H_2_O. PCR reactions were carried out using a Stratagene Mx3000P Real-Time PCR system (Agilent Technologies, USA) with the following steps: pre-denaturation at 95 °C for 2 min, followed by 40 cycles of 94 °C for 20 s, 58 °C for 20 s, and 72 °C for 30 s. Each reaction was performed three times. Fold differences in cDNA level relative to the GAPDH level were calculated using the 2^−ΔΔCt^ method. The following primers were used: IL-2Rα sense, 5′-AAATGACCCACGGGAAGAC-3′; IL-2Rα antisense, 5′-TTGTGACGAGGCAGGAAGT-3′; LMP1 sense, 5′-CAACAACGGCAAGACTCCC-3′; LMP1 antisense, 5′-CCTCAAAGAAGCCACCCTC-3′).

### Measurement of sIL-2Rα in culture supernatant

NK-92, SNK-6 and NK-92 transduced with lentivirus encoding LMP1, and NK-92 and SNK-6 transduced with lentivirus encoding IL-2Rα or negative control lentivirus were centrifuged at 382×*g* for 5 min. sIL-2Rα concentration in the supernatant was measured using a sandwich enzyme-linked immunosorbent assay (Fine Biological Technology, Wuhan, China).

### Cell proliferation and cytotoxicity assay

Cell proliferation was assessed using the Cell Counting kit-8 (CCK-8; Dojin, Tokyo, Japan). For cytotoxicity assay, SNK-6 cells were exposed to doxorubicin, gemcitabine, or asparaginase of varying concentrations for 24 or 48 h prior to CCK-8 assay. Optical density was measured at a wavelength of 450 nm using a Multiskan microplate reader (Thermo Fisher Scientific, Waltham, MA, USA). Relative fold drug resistance was calculated using IC_50_ values.

### Analysis of cell cycle distribution and apoptosis

Flow cytometry was used to determine cell cycle distribution and detect apoptosis. Upon 85% confluence, culture medium was removed and cells were suspended, centrifuged and fixed in precooled 70% ethanol for 1 h. The suspension was centrifuged again, the supernatant was removed, and the cells were washed with ice-cold PBS and stained with propidium iodide (PI; 50 μg/ml, Sigma-Aldrich, St. Louis, MO, USA) in the presence of RNase A (100 μg/ml; Fermentas^®^, Shanghai, China). The suspension was passed through a 300-mesh filter, and DNA content of stained nuclei was analyzed using a BD FACS Calibur flow cytometer (BD Biosciences, San Diego, CA, USA). Each experiment was performed in triplicate.

Apoptosis was analyzed using the Annexin V-APC/7-AAD Apoptosis Detection Kit (Lianke Bio, Hangzhou, China). The percentage of apoptotic cells was determined by flow cytometry on a BD FACS Calibur flow cytometer. All experiments were performed in triplicate.

### Statistical analysis

Results are expressed as mean ± SD. Statistical analysis was performed using SPSS 17.0 (IBM, Chicago, IL, USA). Inter-group differences were assessed for significance using Student’s *t*-test. Differences were defined as statistically significant at *P* < 0.05 (2-sided).

## Results

### Expression of IL-2Rα is higher in NKTCL cells than in natural killer cells

**Fig. 1:**
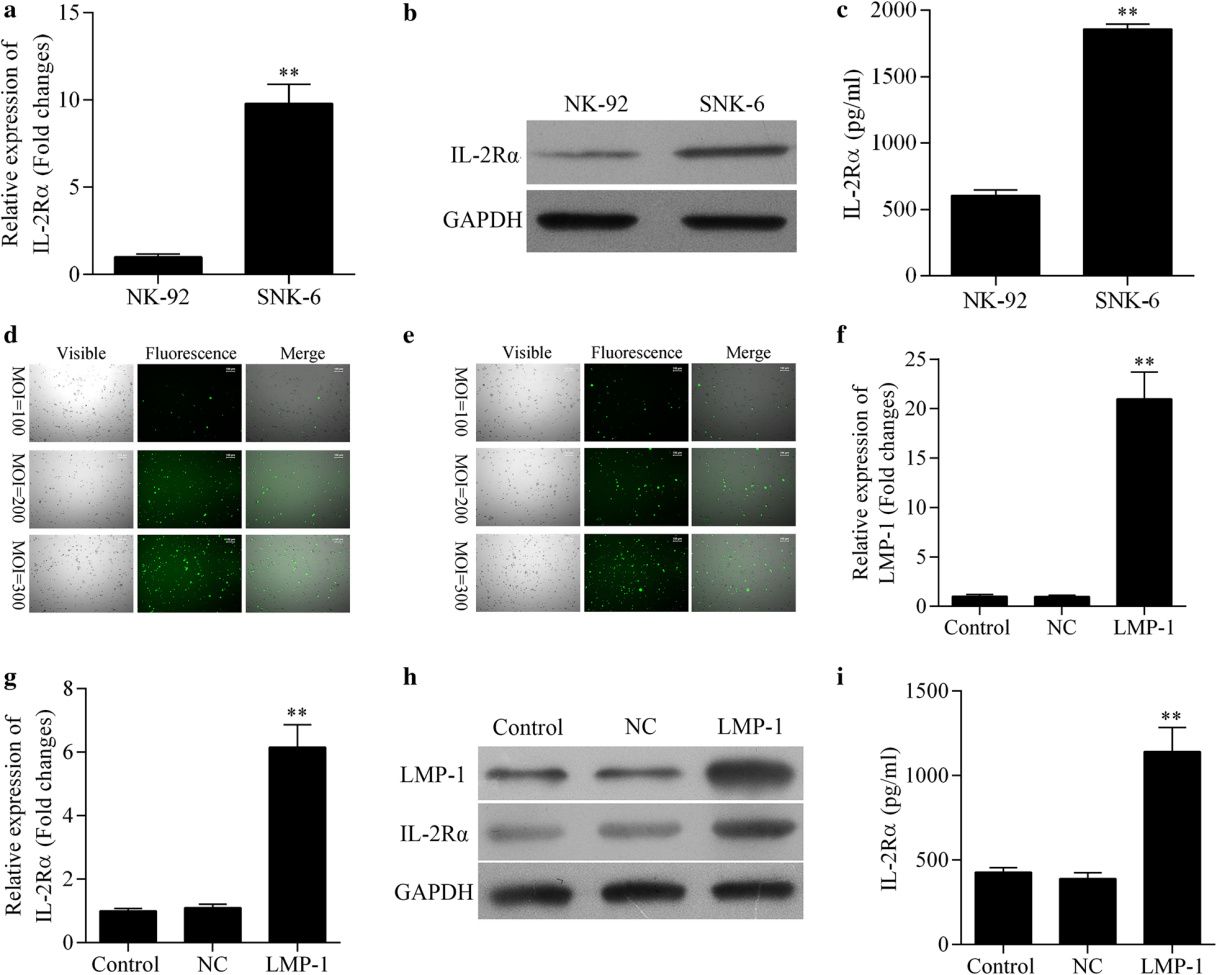
**a**–**c** Analysis of NK-92 and SNK-6 cell lines in terms of levels of **a** IL-2Rα mRNA by quantitative real-time PCR, **b** IL-2Rα protein by Western blot, and **c** soluble IL-2Rα protein in culture supernatant by ELISA. **P < 0.01 vs NK-92 cells. **d**, **e** Efficiency of NK-92 cell infection with **d** lentivirus encoding LMP1 or **e** negative control lentivirus at multiplicities of infection (MOIs) 100, 200, or 300. **f**–**i** Analysis of NK-92 cells (control) and NK-92 cells transduced with lentivirus encoding LMP1 (LMP1) or negative control lentivirus (NC) in terms of levels of **f** LMP1 and **g** IL-2Rα mRNA by quantitative real-time PCR, **h** LMP1 and IL-2Rα proteins by Western blot, and **i** soluble IL-2Rα protein in culture supernatant by ELISA. **P < 0.01 vs control or NC

IL-2Rα expression was significantly higher in SNK-6 cells than in NK-92 cells at both the mRNA (Fig. 1a) and protein levels (Fig. 1b). Similarly, the level of sIL-2Rα in culture supernatant was significantly higher in SNK-6 cells (Fig. 1c).

### LMP1 acts via the MAPK/NF-κB pathway to up-regulate IL-2Rα in NK-92 cells

Lentivirus encoding LMP1 and negative control lentivirus infected cells efficiently at a multiplicity of infection of 300 (Fig. 1d, e). Western blot analysis revealed significantly higher LMP1 expression in NK-92 cells expressing lentivirus-encoded LMP1 at the mRNA (Fig. 1f) and protein levels (Fig. 1h) than in negative control lentivirus vector control. Similar results were observed with IL-2Rα (Fig. 1g, h) as well as sIL-2Rα in culture supernatant (Fig. 1i).

**Fig. 2:**
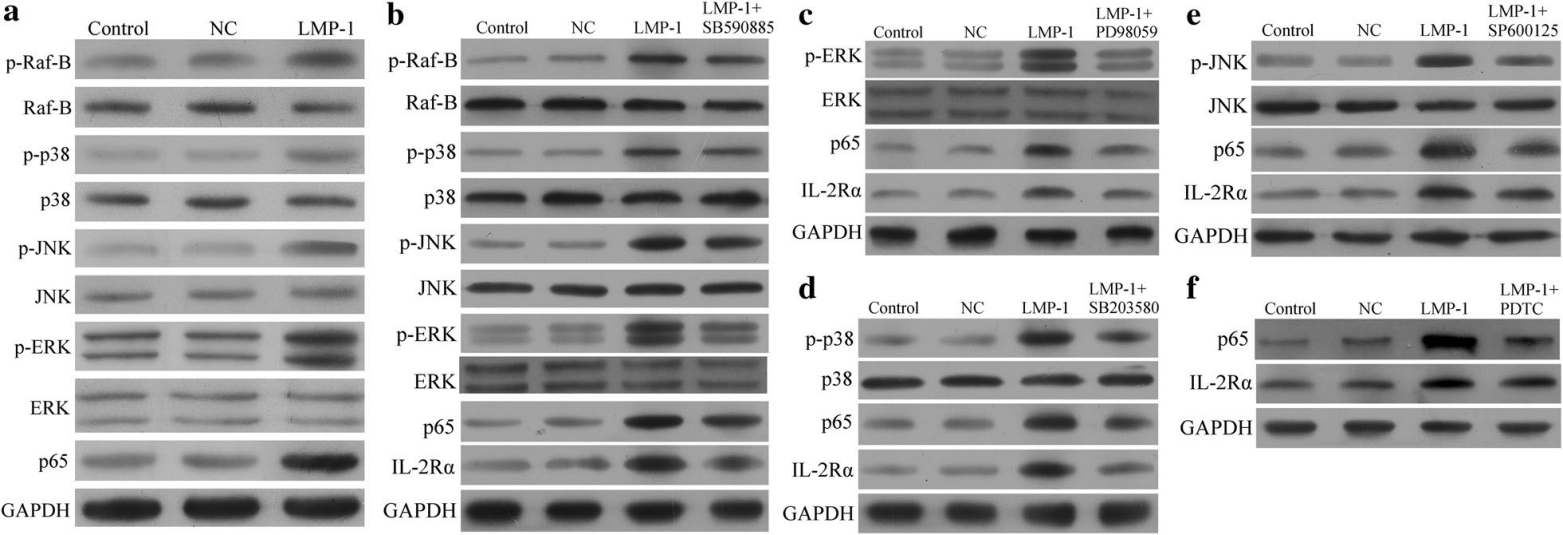
LMP1 up-regulated IL-2Rα expression through the MAPK/NF-κB pathway in NK-92 cells. **a** NK-92 cells transduced with LMP1-encoding lentivirus led to higher levels of p-Raf-B, p-p38, p-JNK, p-ERK, and p65 than transduction with NC lentivirus. **b**–**f** NK-92 cells transduced with LMP1-encoding lentivirus were treated for 1 h with the following selective inhibitors of proteins in the MAPK/NF-κB pathway, leading to IL-2Rα down-regulation: **b** 0.1 μM SB590885 (B-Raf inhibitor), **c** 20 μM PD98059 (ERK inhibitor), **d** 10 μM SB203580 (p38 inhibitor), **e** 20 μM SP600125 (JNK inhibitor), and **f** 100 μM pyrrolidine dithiocarbamate (PDTC; NF-κB inhibitor)

**Fig. 3:**
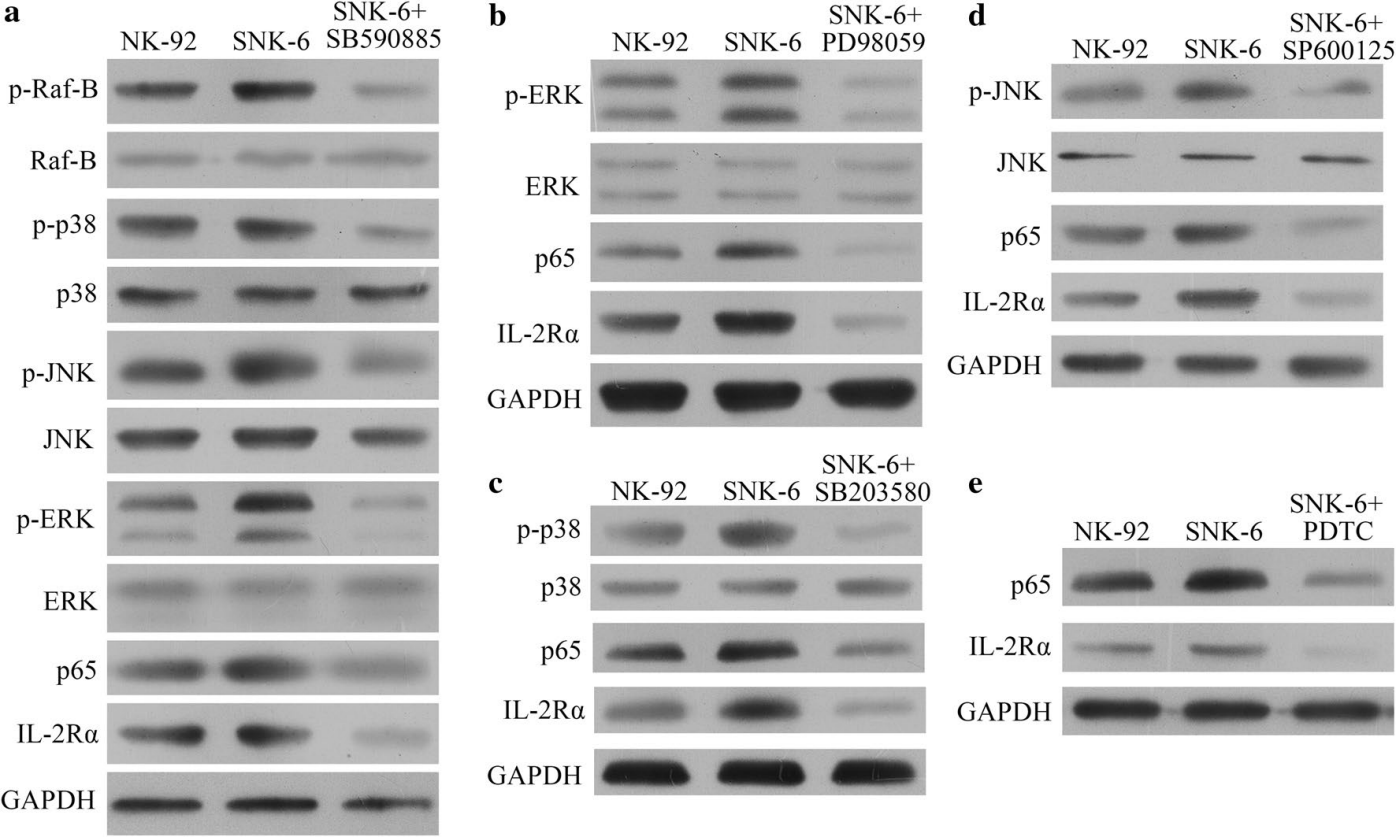
Treating SNK-6 cells constitutively expressing LMP1 for 1 h with the following selective inhibitors of proteins in the MAPK/NF-κB pathway down-regulated IL-2Rα: **a** 0.1 μM SB590885 (B-Raf inhibitor), **b** 20 μM PD98059 (ERK inhibitor), **c** 10 μM SB203580 (p38 inhibitor), **d** 20 μM SP600125 (JNK inhibitor), or **e** 100 μM pyrrolidine dithiocarbamate (PDTC, NF-κB inhibitor)

Up-regulation of these proteins correlated with increases in the levels of several proteins in the MAPK/NF-κB pathway: p-Raf-B, p-p38, p-JNK, p-ERK, and p65 (Fig. 2a). When NK-92 cells expressing lentivirus-encoded LMP1 were treated with the following selective inhibitors of proteins in the MAPK/NF-κB pathway, IL-2Rα was down-regulated: SB590885, which inhibits B-Raf (Fig. 2b); PD98059, which inhibits ERK (Fig. 2c); SB203580, which inhibits p38 (Fig. 2d); SP600125, which inhibits JNK (Fig. 2e); and pyrrolidine dithiocarbamate (PDTC), which inhibits NF-κB (Fig. 2f). Similarly, inhibitors of the MAPK/NF-κB pathway remarkably reduced expression of IL-2Rα in SNK-6 cells constitutively expressing LMP1 (Fig. 3).

### IL-2Rα overexpression promotes proliferation of NK-92 and SNK-6 cells

**Fig. 4:**
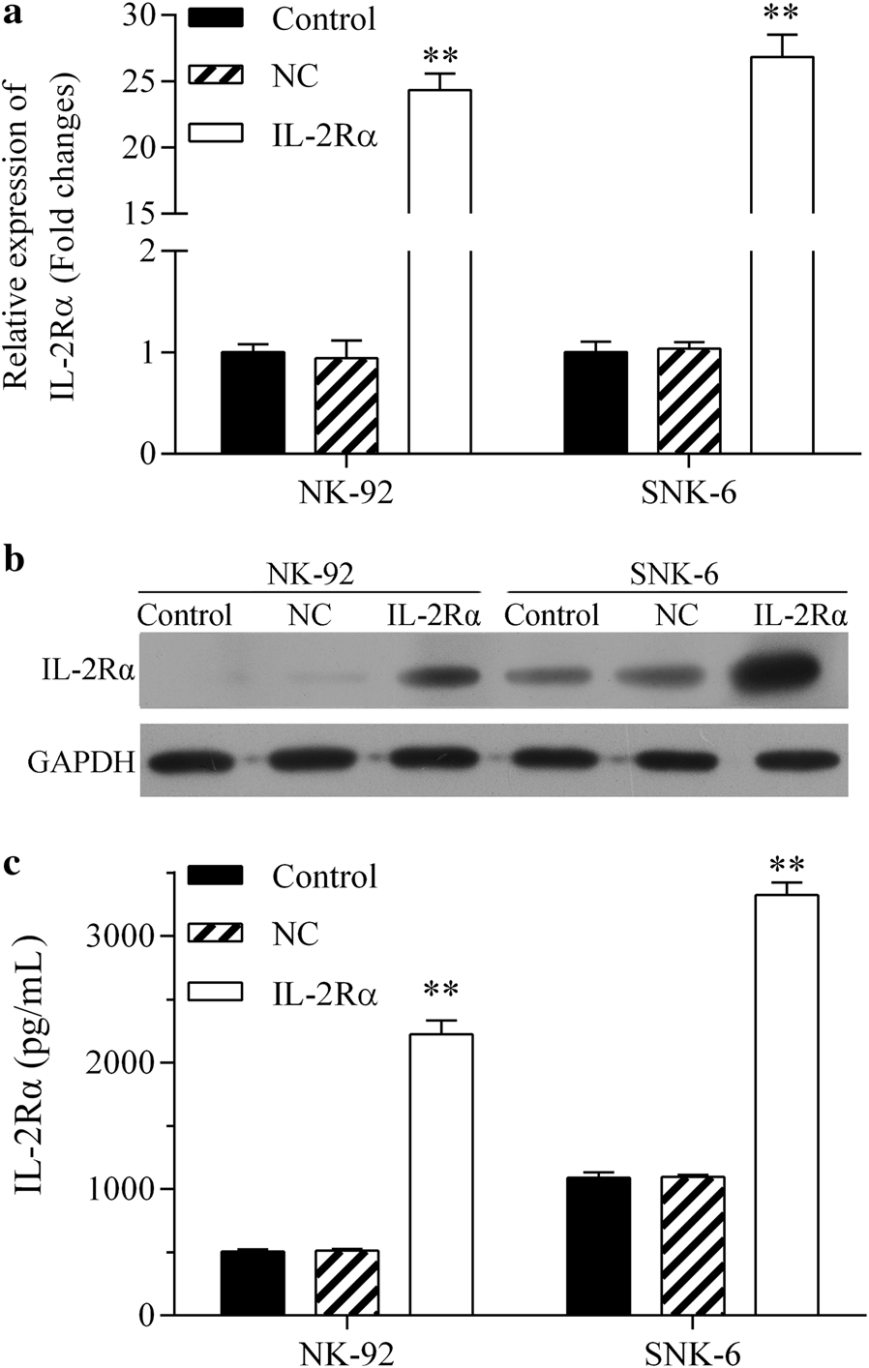
Non-transduced NK-92 and SNK-6 cells (control) as well as the same cell lines transduced with lentivirus encoding IL-2Rα or negative control lentivirus (NC) were analyzed in terms of levels of **a** IL-2Rα mRNA by quantitative real-time PCR, **b** IL-2Rα protein by Western blot, and **c** soluble IL-2Rα protein in culture supernatant by ELISA. **P < 0.01 vs control or NC

**Fig. 5:**
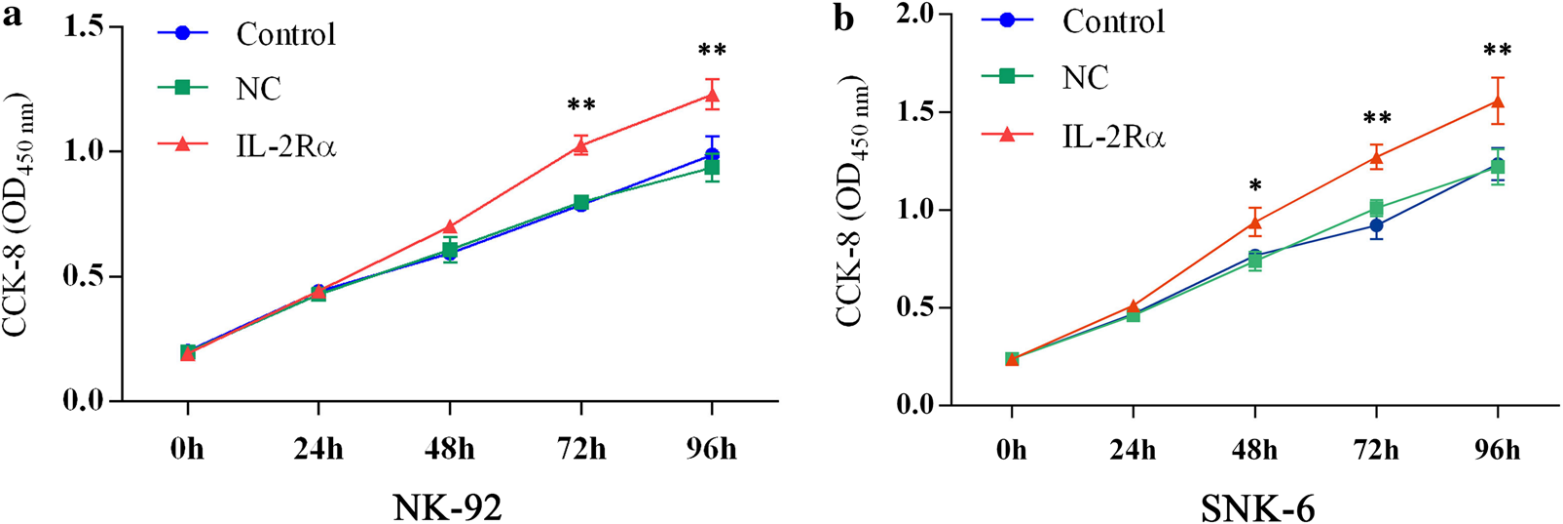
Growth curves of **a** NK-92 and **b** SNK-6 cells. Numbers of cells were determined at 24-h intervals after infection with lentivirus encoding IL-2Rα or negative control (NC) lentivirus. Data are mean ± SD. *P < 0.05, **P < 0.01 vs uninfected cells (control) or cells infected with NC lentivirus

**Fig. 6:**
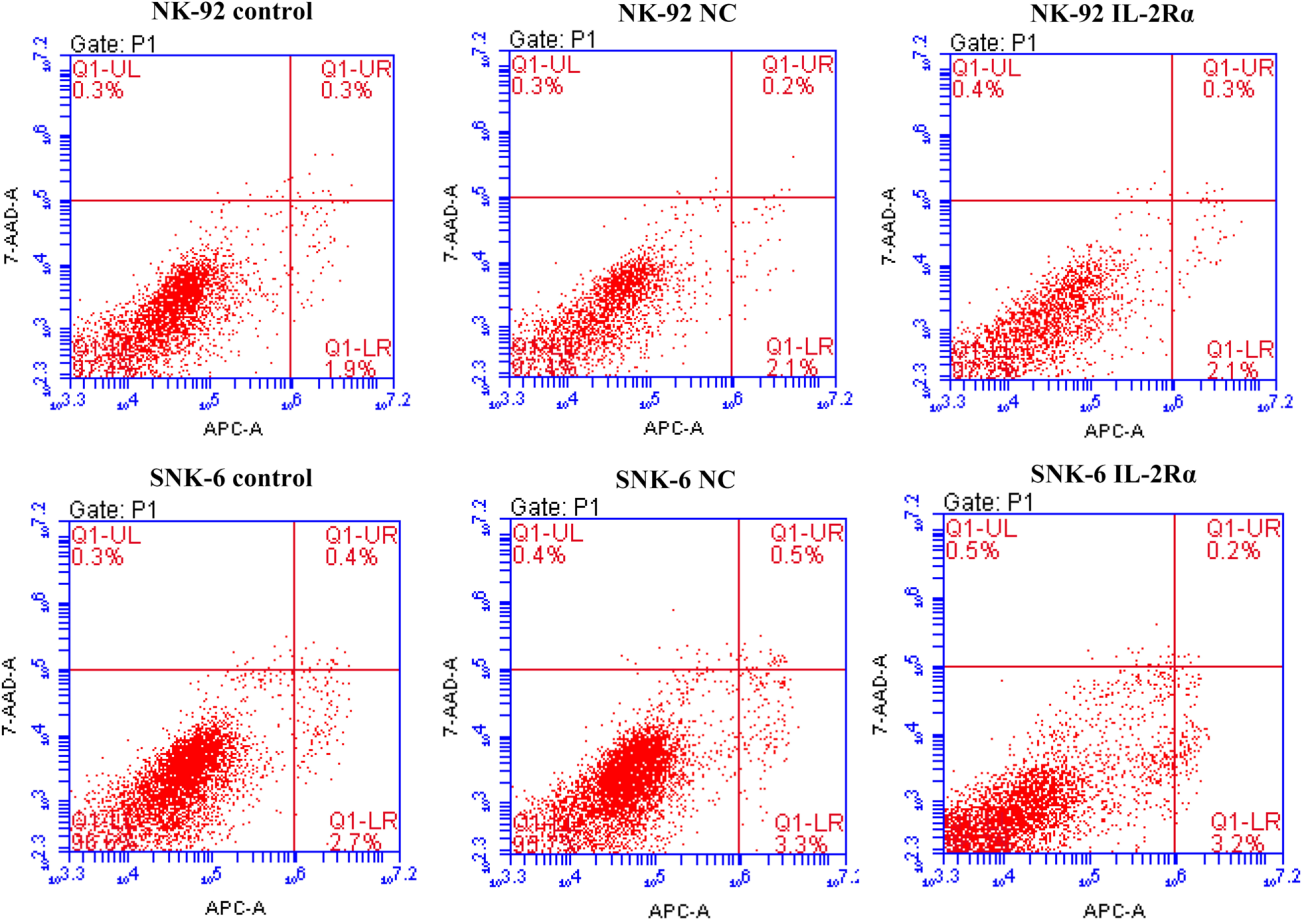
Apoptosis assay of NK-92 and SNK-6 cells transduced with lentivirus-encoded IL-2Rα or negative control (NC) lentivirus vector. Cells were collected and resuspended in binding buffer containing Annexin V-PE and 7-AAD, then processed for flow cytometry. In each box, the left lower region indicates viable cells negative for 7-AAD and Annexin V-PE; the left upper region indicates damaged cells; the right lower region indicates early-stage apoptotic cells positive for Annexin V-PE but negative for 7-AAD; and the right upper region indicates late-stage apoptotic or dead cells positive for Annexin V-PE and 7-AAD. Numbers indicate the percentage of cells in each region. Results are representative of three independent experiments

The IL-2Rα expression was significantly higher in cells expressing lentivirus-encoded IL-2Rα at the mRNA (Fig. 4a) and protein levels (Fig. 4b) in comparison to the negative lentivirus control. Cells expressing lentivirus-encoded IL-2Rα also secreted higher amount of sIL-2Rα into the supernatant (Fig. 4c), and had higher rate of proliferation (Fig. 5a, b). IL-2Rα overexpression did not significantly alter the percentage of apoptotic cells (Fig. 6).

**Fig. 7:**
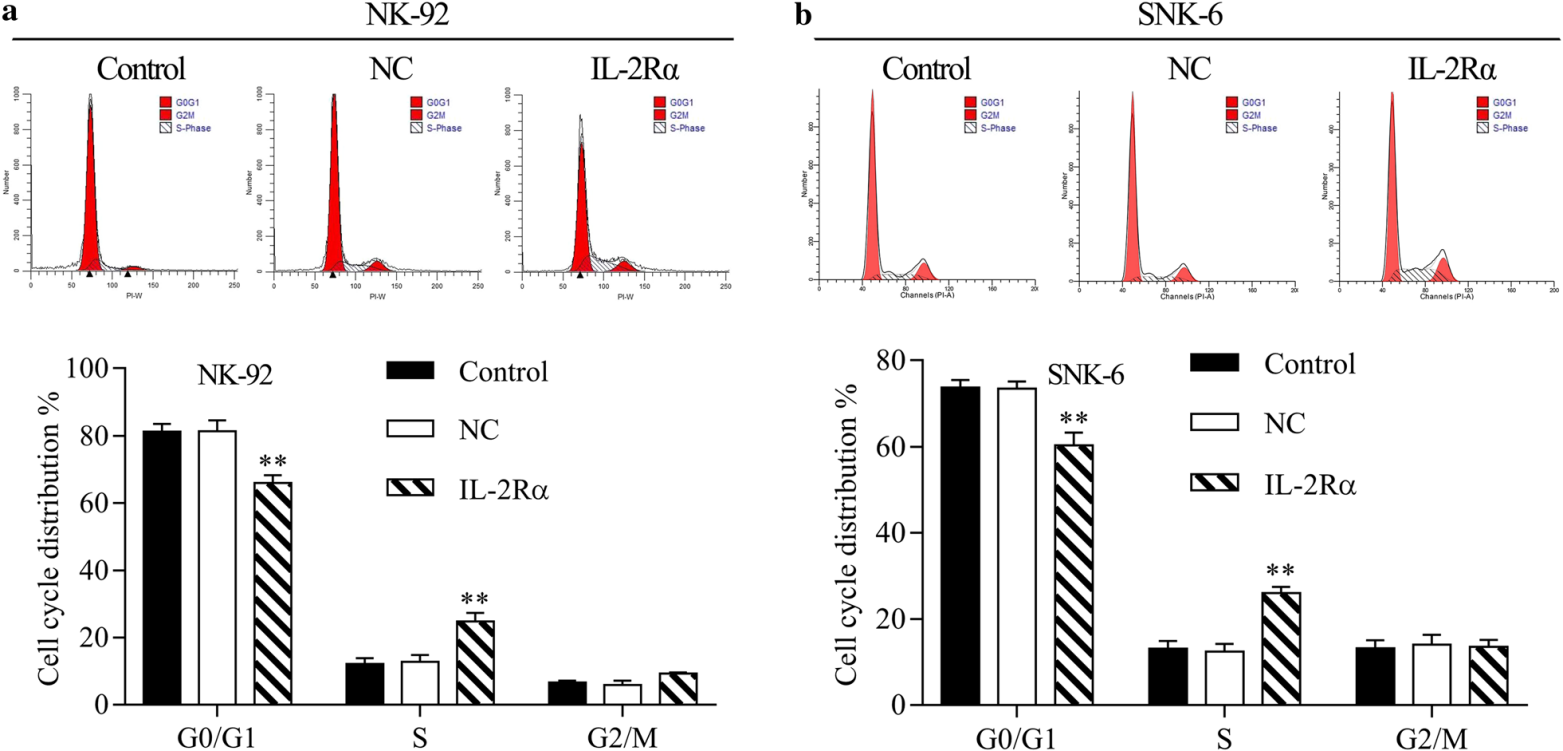
Cell cycle distribution of **a** NK-92 and **b** SNK-6 cells expressing lentivirus-encoded IL-2Rα. **P < 0.01 vs uninfected cells (control) or cells infected with NC lentivirus

IL-2Rα overexpression decreased the percentage of cells in the G0/1 phase and increased the percentage in the S phase (Fig. 7). These changes were caused by increases in protein levels of cyclin A1, A2, B1, D, CDK1, and CDK4 (Additional file 1: Figure S1).

### IL-2Rα overexpression mediates chemoresistance in SNK-6 cells

**Fig. 8:**
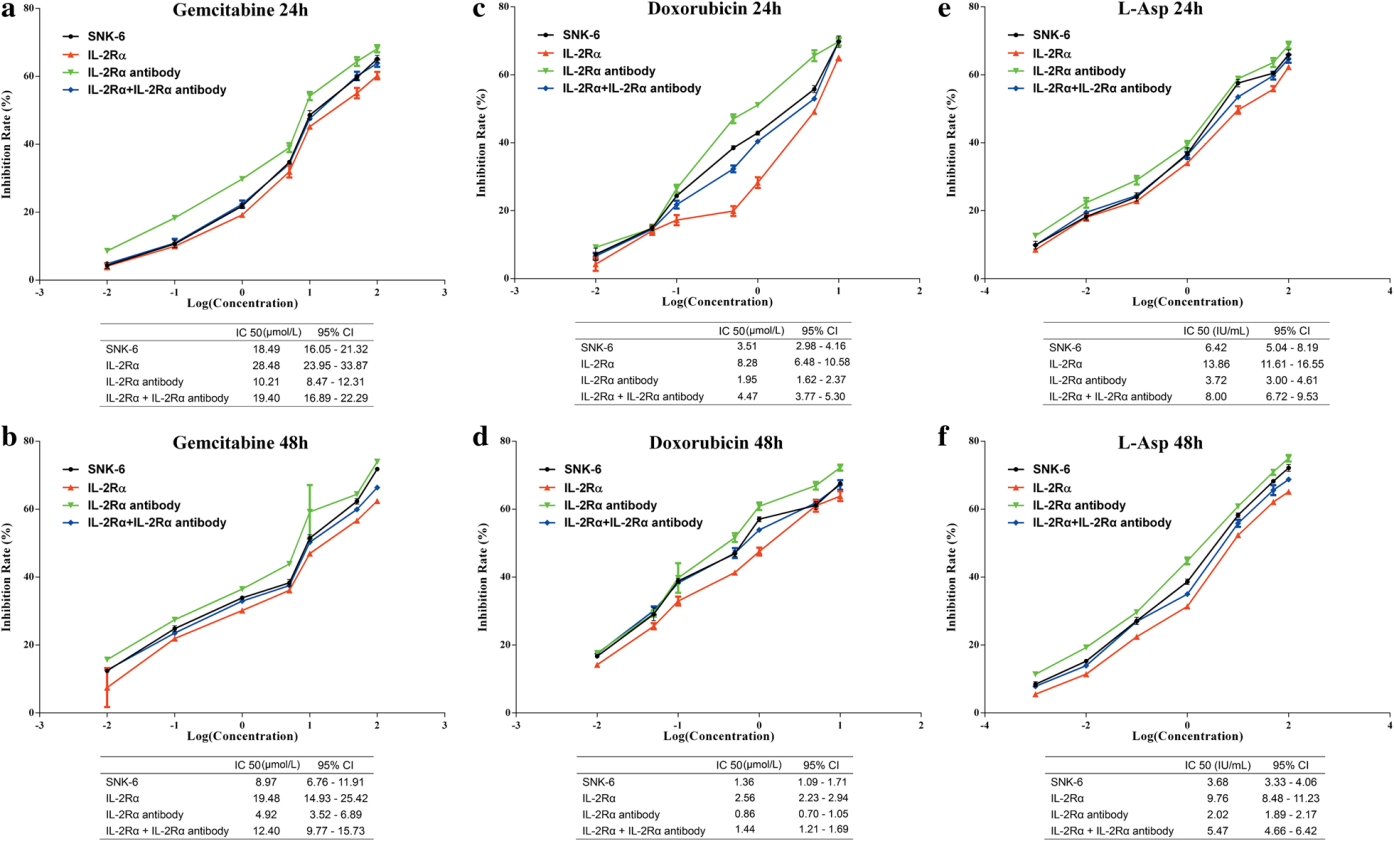
Influence of IL-2Rα and its antibody on sensitivity to **a**, **b** gemcitabine, **c**, **d** doxorubicin, and **e**, **f** l-asparaginase (l-Asp) in SNK-6 cells. Cells overexpressed with IL-2Rα were treated with anti-IL-2Rα antibody (0.2 μg/ml) and different concentrations of one of the three drugs. Cell viability was determined by CCK-8 assay. IC_50_ values are presented as mean (95% confidence interval)

IL-2Rα overexpression in SNK-6 cells resulted in significantly higher IC_50_ values against gemcitabine, doxorubicin, and l-Asp (Fig. 8). Addition of anti-IL-2Rα antibody (0.2 μg/ml; Beijing Shuanglu Pharmaceutical, Beijing, China) reduced all three IC_50_ values.

### Targeting of IL-2Rα to treat relapsed/refractory NKTCL

A 42-year-old man with stage IV NKTCL who relapsed after six cycles of GELOX (gemcitabine, oxaliplatin, and asparaginase) and who did not respond to two cycles of EPOCH (etoposide, prednisone, vincristine, cyclophosphamide, and adriamycin) and two cycles of SMILE (dexamethasone, methotrexate, ifosphamide, asparaginase, and etoposide) was then given two cycles of pegaspargase and anti-IL-2Rα antibody basiliximab (2500 IU/m^2^ pegaspargase given on day 1 + 20 mg basiliximab given on day 1 and 8, repeated every 3 weeks). This last treatment achieved partial remission and its toxicity was tolerable (Additional file 2: Figure S2). Levels of Epstein–Barr virus DNA in plasma decreased from 4.2 × 10^3^ copies/mL to 0.

## Discussion

NKTCL is a rare, highly aggressive hematologic malignancy. The frequency with which the disease is resistant to conventional chemotherapy (e.g., CHOP and EPOCH) has made radiotherapy the primary therapy [5, 8, 21, 22]. Although novel regimens incorporating l-Asp or pegaspargase have significantly improved outcomes, 20%–40% of patients still experience treatment failure [6, 7, 9, 10]. Therefore, there is an urgent need to develop new biological and genetic biomarkers to predict prognosis and guide therapy.

In previous clinical work, our group reported that sIL-2Rα levels in serum were significantly elevated in NKTCL, and that these levels correlated with chemotherapy response and prognosis [19]. The present study extends and deepens that work by describing a pathway by which IL-2Rα overexpression promotes NKTCL tumor growth, and by showing the potential of anti-IL-2Rα antibody therapy. Here we describe several major findings: (1) LMP1 acts via the MAPK/NF-κB pathway to up-regulate IL-2Rα in NKTCL, (2) IL-2Rα overexpression promotes NKTCL cell proliferation and cell cycle progression without affecting apoptosis, and (3) IL-2Rα overexpression correlates positively with chemoresistance in NKTCL cells, and anti-IL-2Rα antibody can restore chemosensitivity.

Elevation of various cytokines has been observed in both non-Hodgkin and Hodgkin lymphoma [23–25]. IL-2 acts as a potent immunomodulator and activates many immune cells, including antigen-specific T cells, B cells, and natural killer cells [12]. Elevated expression of the IL-2Rα subunit of the IL-2 receptor has been reported in several types of malignancy, including NKTCL [26, 27]. The high affinity of IL-2Rα for IL-2 led us to hypothesize that IL-2Rα up-regulation may promote tumor cell proliferation and progression. Consistent with this idea, a preliminary study of NKTCL patients showed that in 53.8% of cases, IL-2Rα was expressed on the tumor surface (data not shown). In the present study, IL-2Rα expression was significantly stronger in NKTCL cells than in natural killer cells, and more sIL-2Rα was present in supernatants of NKTCL cultures.

We provide here evidence that IL-2Rα up-regulation in NKTCL may be mediated by the Epstein–Barr viral protein LMP1. The Epstein–Barr virus plays a pivotal role in NKTCL pathogenesis, and LMP1 regulates several cytokines and cytokine receptors in malignancies associated with the Epstein–Barr virus [28–30]. We found that LMP1 expression in NK-92 cells up-regulated IL-2Rα and sIL-2Rα levels, which was reversed by selective inhibitors of the MAPK/NF-κB pathway. This is consistent with previous work showing that LMP-1 acts via the NF-κB pathway to up-regulate IL-2Rα in two other lymphomas associated with Epstein–Barr virus, Hodgkin’s lymphoma and Burkitt’s lymphoma [31].

Elevated levels of sIL-2R predict inferior outcomes in several types of lymphoma, including B-cell and T-cell non-Hodgkin lymphomas [18, 32, 33]. In some hematological neoplasms, such as hairy-cell leukemia and adult T-cell leukemia, sIL-2Rα is released by tumor cells constitutively expressing IL-2Rα [34, 35]. Therefore, the serum level of sIL-2Rα may reflect tumor burden and disease activity. The intracellular domain of IL-2Rα is too short to elicit signal transduction on its own, but IL-2Rα promotes IL-2 recycling back to the cell surface, creating an IL-2 reservoir at the surface, thereby potentiating its activity [36]. This activates oncogenic pathways such as JAK-STAT, MAPK, and PI3 K, with mitogenic and anti-apoptotic effects [37, 38]. At the same time, sIL-2Rα binds soluble sIL-2, and the complex activates tumor-friendly Treg cells rather than antitumor T cells [32]. In the present study, we report for the first time that IL-2Rα overexpression promotes NKTCL cell proliferation and cell cycle progression without affecting apoptosis. This is consistent with previous reports that IL-2 activates and promotes natural killer cell proliferation [39], and further work should clarify the mechanism(s) involved.

Resistance to chemotherapy is the major cause of treatment failure in NKTCL, especially in advanced disease [40]. While overexpression of multi-drug resistance genes may contribute to this chemoresistance [4], our previous work showed that elevated serum levels of sIL-2Rα correlated with significantly inferior response rate to chemotherapy (mainly CHOP or EPOCH regimens) [19]. Consistent with this previous work, we report here that IL-2Rα overexpression in NKTCL cells led to chemoresistance in vitro to gemcitabine, doxorubicin, and l-Asp. As further evidence for the involvement of IL-2Rα, this chemoresistance was reversed by anti-IL-2Rα antibody. This suggests the potential of targeting IL-2Rα for treating NKTCL. Indeed, we combined the anti-IL-2Rα antibody basiliximab with pegaspargase to successfully treat a patient with relapsed/refractory NKTCL. Both basiliximab and the humanized monoclonal anti-IL-2Rα antibody daclizumab have been used to treat autoimmune diseases such as multiple sclerosis as well as prevent transplant rejection. We plan to conduct a prospective phase 2 clinical trial to evaluate the efficacy of combination of basiliximab and pegaspargase in patients with relapsed/refractory NKTCL [41].

Our results should be interpreted with caution given several limitations. We did not investigate which IL-2-associated signaling pathways may help mediate the tumor-promoting effects of IL-2Rα in NKTCL, nor did we examine the immunomodulatory effects of IL-2Rα in NKTCL. The role of serum sIL-2Rα in lymphomagenesis needs to be further investigated. Thorough study of these questions will improve our understanding of the role of IL-2Rα in the pathogenesis and progression of NKTCL.

## Conclusions

In NKTCL, LMP-1 acts via the NF-κB pathway to up-regulate IL-2Rα, and promotes tumor growth. Further studies are warranted to understand how IL-2Rα exerts its oncogenic effects and to explore IL-2Rα as a therapeutic target.

## Additional files

**Additional file 1: Figure S1.** Levels of several cell cycle proteins were increased in SNK-6 cells when IL-2Rα was overexpressed, as detected by Western blot. Control samples came from uninfected cells, while negative control samples came from cells infected with NC lentivirus.

**Additional file 2: Figure S2.** Positron emission tomography-computed tomography of a 42-year-old man with refractory NKTCL before and after treatment with anti-IL-2Rα antibody (basiliximab) and pegaspargase. Before treatment, diffuse infiltration of lymphoma with high metabolic activity was observed (*right panel*). After two cycles of treatment, partial remission was observed (*left panel*).

## Abbreviations

NKTCL: natural killer/T-cell lymphoma
IL-2Rα: IL-2 receptor α
l-Asp: l-asparaginase
IL-2: interleukin-2
LMP1: latent membrane protein-1
