# Supplementary figures and images for: IL-2Rα up-regulation is mediated by latent membrane protein 1 and promotes lymphomagenesis and chemotherapy resistance in natural killer/T-cell lymphoma
Source: Cancer Commun (Lond). 2018 Oct 19;38:62. doi: 10.1186/s40880-018-0334-8 (PMC6235395; doi:10.1186/s40880-018-0334-8)

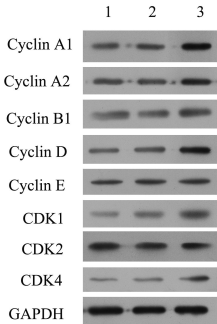

1. Control

2. Negative control

3. IL-2R $\alpha$  overexpression

Supplement: Supplementary file 1 — Additional file 1: Figure S1. Levels of several cell cycle proteins were increased in SNK-6 cells when IL-2Rα was overexpressed, as detected by Western blot. Control samples came from uninfected cells, while negative control samples came from cells infected with NC lentivirus. [file 40880_2018_334_MOESM1_ESM.pdf]

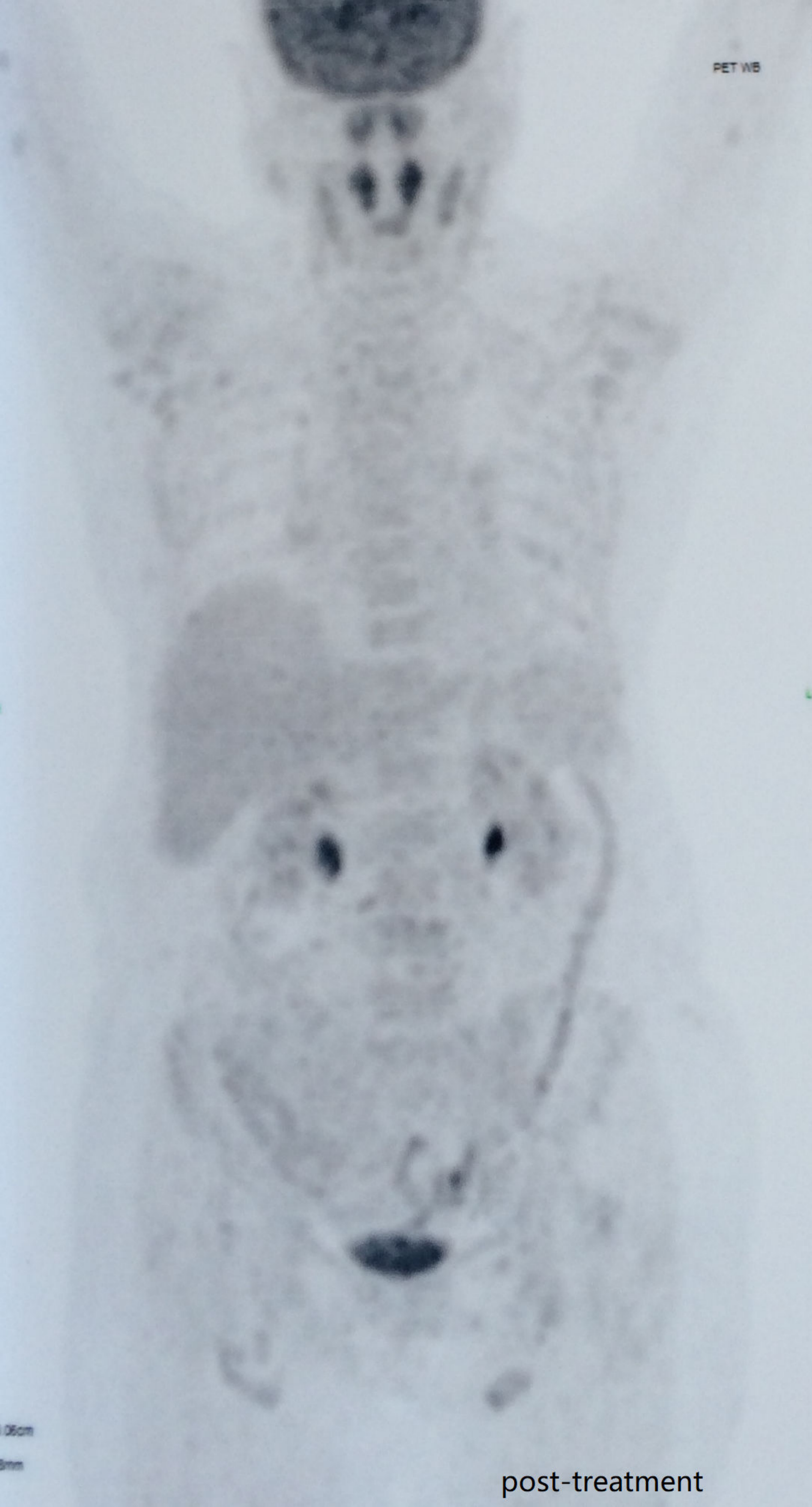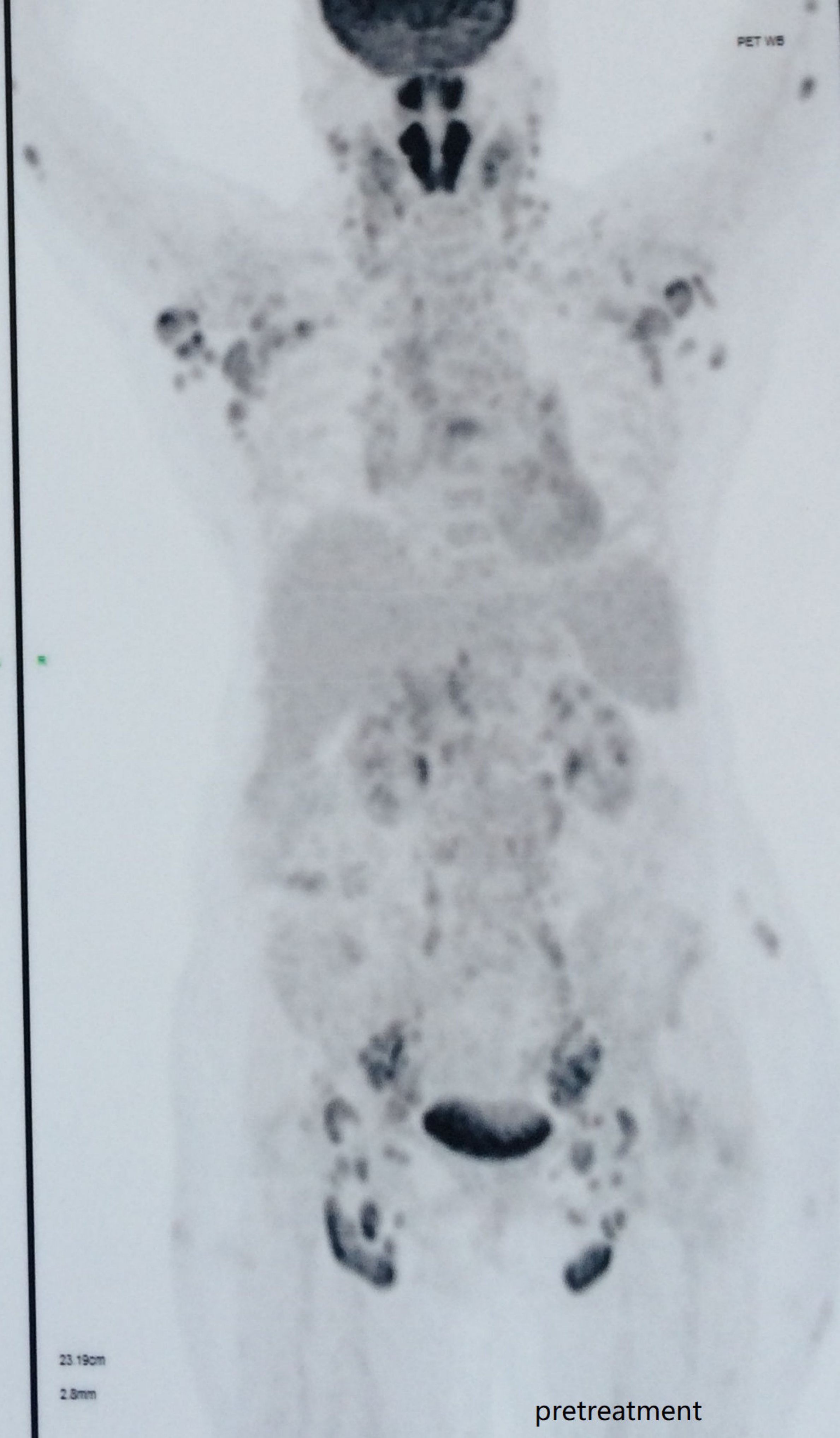

Supplement: Supplementary file 2 — Additional file 2: Figure S2. Positron emission tomography-computed tomography of a 42-year-old man with refractory NKTCL before and after treatment with anti-IL-2Rα antibody (basiliximab) and pegaspargase. Before treatment, diffuse infiltration of lymphoma with high metabolic activity was observed (right panel). After two cycles of treatment, partial remission was observed (left panel). [file 40880_2018_334_MOESM2_ESM.pdf]
